# How does lean work in emergency care? A case study of a lean-inspired intervention at the Astrid Lindgren Children's hospital, Stockholm, Sweden

**DOI:** 10.1186/1472-6963-12-28

**Published:** 2012-02-01

**Authors:** Pamela Mazzocato, Richard J Holden, Mats Brommels, Håkan Aronsson, Ulrika Bäckman, Mattias Elg, Johan Thor

**Affiliations:** 1 Medical Management Centre, Karolinska Institutet, Berzelius väg 3, Stockholm, Sweden; 2 Department of Medicine and Biomedical Informatics, Vanderbilt University School of Medicine, 733 Medical Arts Building, 1211 21st Avenue S, Nashville, TN, USA; 3 Department of Management and Engineering, Linköping University, Linköping, Sweden; 4 Astrid Lindgren Children's Hospital/Karolinska University Hospital, Karolinska vägen, Stockholm, Sweden

## Abstract

**Background:**

There is growing interest in applying lean thinking in healthcare, yet, there is still limited knowledge of how and why lean interventions succeed (or fail). To address this gap, this in-depth case study examines a lean-inspired intervention in a Swedish pediatric Accident and Emergency department.

**Methods:**

We used a mixed methods explanatory single case study design. Hospital performance data were analyzed using analysis of variance (ANOVA) and statistical process control techniques to assess changes in performance one year before and two years after the intervention. We collected qualitative data through non-participant observations, semi-structured interviews, and internal documents to describe the process and content of the lean intervention. We then analyzed empirical findings using four theoretical lean principles (Spear and Bowen 1999) to understand how and why the intervention worked in its local context as well as to identify its strengths and weaknesses.

**Results:**

Improvements in waiting and lead times (19-24%) were achieved and sustained in the two years following lean-inspired changes to employee roles, staffing and scheduling, communication and coordination, expertise, workspace layout, and problem solving. These changes resulted in improvement because they: (a) standardized work and reduced ambiguity, (b) connected people who were dependent on one another, (c) enhanced seamless, uninterrupted flow through the process, and (d) empowered staff to investigate problems and to develop countermeasures using a "scientific method". Contextual factors that may explain why not even greater improvement was achieved included: a mismatch between job tasks, licensing constraints, and competence; a perception of being monitored, and discomfort with inter-professional collaboration.

**Conclusions:**

Drawing on Spear and Bowen's theoretical propositions, this study explains how a package of lean-like changes translated into better care process management. It adds new knowledge regarding how lean principles can be beneficially applied in healthcare and identifies changes to professional roles as a potential challenge when introducing lean thinking there. This knowledge may enable health care organizations and managers in other settings to configure their own lean program and to better understand the reasons behind lean's success (or failure).

## Background

Accident and emergency departments (A&Es) all over the world are challenged with problems of overcrowding and excessive waiting times [1-3]. Overcrowding and delays correlate with increased patient mortality, decreased patient and staff satisfaction, and inefficient use of resources [4,5]. Moreover, as A&Es are considered to be the heart of hospitals [6], problems there may affect the whole organization.

Process and flow problems are factors that contribute to delays and overcrowding. Indeed, health care delivery has traditionally been organized around specialties and professional groups that address patients' problems one at a time (function-based organization), rather than around the entirety of each patient's needs [7-9]. Hoping to overcome the limitations of function-based organization, many healthcare organizations are adopting approaches such as lean thinking to better integrate health care delivery. The term lean thinking (hereafter referred to as "lean") is based on a production philosophy originally developed by Toyota Motor Corporation. It consists of principles and practices that focus on minimizing the total time and resources needed to produce and supply goods or services to a customer, thus increasing efficiency. Reductions in time and resource use are achieved by focusing on value-adding steps and eliminating non-value-adding steps in the production process [10,11].

Literature reviews show that lean has been applied with success to a wide range of clinical situations [12-15]. In particular, Holden's recent review of lean applications in A&Es shows that lean can contribute to decreases in waiting times, length of stay, and the proportion of patients leaving without being seen [16]. As a whole, lean in health care is nevertheless at an early stage of development [12,14,17]. Most published examples of lean in healthcare focus on the use of particular tools, such as process mapping, to achieve short-term improvements [13,14,17]. Little is typically done to enable structured problem solving involving frontline workers and management [14,18] and there are only a few cases in which lean interventions are integrated into an organizational-wide strategy [13,14,17]. Virginia Mason Medical Center and ThedaCare are some of the exceptions [19,20].

Research on lean is also limited. Studies of lean often lack explicitly stated and appropriate research designs, appropriate statistical tests, and outcome measures [12,14,21]. There is also a dominance of studies reporting successful lean interventions [12,14] whereas little has been reported about failed attempts or barriers to application.

In sum, lean attracts interest as an approach to healthcare improvement, particularly in A&E settings, but further research is required to understand its feasibility and effectiveness. In addition to more rigorous tests of *whether *(or to what extent) lean "works" in healthcare [22], research is needed to fill the present knowledge gap regarding *how *and *why *lean may work in healthcare. Without such knowledge, it is difficult to design lean interventions that yield improvements with the least amount of necessary effort and resources. To generate such understanding, we undertook an in-depth case study of lean-inspired improvement efforts at a pediatric A&E in Sweden. By examining clinical operations and performance before and during the intervention, as well as the associated improvement process, we aimed to unpack how and why such a lean application may work.

## Methods

### Setting

The pediatric A&E in this study is part of the Astrid Lindgren Children's Hospital, one of seven divisions within the Karolinska University Hospital, in Sweden. The Karolinska University Hospital is a publicly funded and owned tertiary care center with over 100,000 admissions annually and over 15,000 employees. It is located in Stockholm and serves a population of 2 million inhabitants. In 2007, under external pressure and directives of the hospital board to improve access to care, the hospital management, led by a new CEO, initiated a strategic long-term lean-inspired program to improve care processes (i.e. increase patient value and decrease waste) and working conditions across Karolinska University Hospital. The initiative began with efforts to improve all emergency patient flows which constituted over 60% of all hospital admissions, including the pediatric patient flow at the studied A&E.

The pediatric A&E is the largest of three pediatric A&Es located in the Stockholm metropolitan area. It is the only one with specialized surgical and trauma care accessible by ambulance and helicopter. A "first line" pediatric emergency center with general practitioners is located next door to the A&E. The A&E has 17 single bed exam rooms, 2 specially equipped rooms for minor surgical procedures, 1 room designed for applying casts, and 1 observation area containing 5 beds. Clinical staff at the A&E include: physician specialists (either pediatricians or pediatric surgeons), residents (residents in pediatrics or residents in family medicine doing their pediatric rotation), registered nurses, and licensed nurse's aides. The A&E includes two separate patient flows: "pediatric" (with children up to 18 years) and "surgical/orthopedic" (with children up to 15 years). Of the 34,870 patients seen in 2008, 20,175 were pediatric patients and 14,695 surgical patients. On average 55 and 40 patients were seen per day at the pediatric and the surgical section, respectively. Patient inflow is characterized by high seasonal and daily volume variation, as well as by great diversity in reasons for patients seeking care. Breathing difficulty and infections are the most common reasons for visiting the pediatric section. Injuries and abdominal pain are the most common reasons for visiting the surgical/orthopedic side.

### Study design and conceptual framework

This is a mixed methods explanatory single case study [23,24] of the lean-inspired improvement efforts at the pediatric section of the A&E described above. From a research perspective, the A&E is an appealing setting for studying lean because A&Es are often targeted, sometimes first, when a hospital "goes lean" [25]. A&Es also exhibit many of the challenges facing contemporary healthcare as a whole, including process and flow problems due to lack of standardization, fragmentation and poor coordination between process steps.

The study includes a quantitative and a qualitative component. The objectives of the quantitative component were to track operational performance changes over time and to compare performance before and after the lean intervention. Performance measures were (a) proportion of patients being discharged from the A&E within 4 hours, and (b) waiting time from triage to first assessment by a doctor.

The objectives of the qualitative component were both to describe the lean intervention and to provide data to help us explain how the intervention worked based on four theoretical lean principles. The four principles were empirically derived by Spear and Bowen [26] to characterize lean operations design and improvement at Toyota (Table 1). According to these principles, lean yields high levels of performance as it: (a) standardizes work and reduces ambiguity, (b) connects people who are dependent on one another, (c) creates seamless, uninterrupted flow of work through the process, and (d) empowers staff to investigate process problems and to develop, test, and implement countermeasures using a "scientific method" [26]. While these principles were originally developed from studies of Toyota-the source for lean-we deemed them useful for our analysis as they explain how lean works, rather than focusing on specific practices or steps to take to implement lean. These principles were not explicitly used by the implementers at the hospital to design the lean intervention; rather, they formed the analytic framework for this study.

**Table 1:** Lean principles and examples of related practices in manufacturing (adapted from [26])

Lean principles	Description of how principles work	Examples of related practices
Standardize work.	"All work shall be highly specified as to content, sequence, timing, and outcome" to reduce variation in how employees do their work.	Standardized job descriptions.

Connect people and machines that are dependent on one another.	"Every customer-supplier connection must be direct, and there must be an unambiguous yes-or-no way to send requests and receive responses." When a worker makes a request for parts or services there is no confusion about who is responsible for providing it, the number of units required or the type of service needed, and the timing of delivery.	*Kan Ba*n cards (an inventory management system that signals when the consumption of an article creates a demand for replenishment), *Jidoka *(equipment that automatically stops when quality problems are detected. This defect is signalled to the responsible operators and managers through display boards).

Create seamless, uninterrupted flow through the process.	"The pathways for every product and service must be simple and direct". All production lines are set up so that every product and service flows along a simple and specified pathway and goods and services do not flow to the next available person or machine but to a specific person or machine.	U-shaped physical layout (a layout that allows workers to move between the different tasks that compose a process in a flexible way), assembly line.

Empower staff to investigate problems with the process and to develop, test, and implement countermeasures using a "scientific method.	Staff members closest to the operations investigate root causes of a problem and develop "countermeasures" that are tested and implemented in accordance with the "scientific method" and under the guidance of a teacher.	Employee empowerment, *Kaizen *events (a team based approach for systematic problem solving).

### Data collection

For the quantitative part of the study, authors PM and UB collected the hospital's weekly averages data of a) the proportion of patients leaving the A&E within 4 hours, b) the waiting time from triage to first A&E physician consultation, and c) the patient volume. All averages included data collected between 08:00 and 16:00 o'clock at the pediatric section of the A&E. Performance and patient volume data were collected for 52 weeks before and 104 weeks after the implementation of lean-inspired changes, which occurred in December 2008.

For the qualitative part, data on the intervention planning phase were collected retrospectively (interviews and documents), while the implementation phase was studied prospectively (observation, interviews, documents). PM conducted 40 hours of non-participant observation [27] by shadowing nurses, residents, and specialists during day and afternoon work shifts. She focused on the workflow, the information flow, and the roles of different actors in the process before and after the intervention. She attended improvement team meetings to gain a deeper understanding of how the intervention was designed and carried out. In addition, she conducted 13 semi-structured interviews with 1 resident, 3 senior physicians, 3 nurses, 1 coach, the director of the Pediatric Division, 2 first-line managers, and 2 administrative staff members. Interviews were carried out during 2008 and 2009 and focused on how work was carried out before and after the intervention, particularly: individuals' work tasks; the way individuals' work related to that of others; factors hindering effective delivery of care; intended and actual process changes; and expected and measured outcomes. PM and UB also collected documents from improvement efforts.

### Data analysis

Analysis of variance (ANOVA) was used to assess differences in performance (percentage of patients leaving the A&E within four hours and waiting time to see a physician) and patient volume between three groups: 52 weeks before lean (pre-lean), 52 weeks after lean (first year post-lean), and 52 weeks follow-up (second year post-lean). Statistical significance was set at 0.05. These outcome variables were all treated as continuous. The assumptions underlying ANOVA were checked using normal probability plots.

The ANOVA was complemented by statistical process control charts, which are often used to analyze patterns of performance over time in a complex system [28-31]. Control charts help to distinguish between common-cause and special-cause variation. Common-cause refers to the natural, inherent and historically given variation of any system. Special-cause variation is characterized by deviations from the natural behavior of the system, such as might be seen following an intervention. We used two types of process control charts, based on recommendations for analyzing different types of data [32]. P-chart analysis was used for the percentage of patients leaving the A&E within four hours, an analysis that allowed us to factor patient inflow into performance variability. An I-chart analysis was used for the time to see a physician. For these analyses, we used two rules to determine whether performance changes signaled special-cause variation (e.g., related to the lean intervention). The first rule indicating performance changes attributable to lean, and the stricter of the two, was whether performance crossed an upper or lower control limit threshold. The control limits were set, as recommended [33], at 3σ, or three standard deviations, on either side of a baseline calculated based on the 52 weeks of performance prior to the lean intervention. The second rule indicating non-chance performance change following lean was if nine consecutive data points fell on the same side of the pre-lean baseline. ANOVA and process control charts were analyzed using MINITAB 16 software.

The qualitative part includes a case description and a case analysis. The case description was carried out in three steps. First, documents were organized in chronological order in an Excel file to reconstruct the implementation process. Second, text format data collected through interviews, observations, and documents were organized and coded in NVivo 8 software to characterize the content of the intervention, i.e. the actual changes carried out, its implementation process, and its effects as perceived by staff members. Third, qualitative data were used to describe the care process before and after the lean intervention from patient arrival to admission or discharge.

The case analysis aimed to explain how and why the intervention worked in the local context as well as to identify its strengths and weaknesses. We first categorized the lean-inspired changes based on Spear and Bowen's principles (Table 1). In addition, taking the explanation building approach described by Yin [23], we compared and adapted these principles (i.e. theoretical statements) to the present context based on our qualitative data [23].

We took several steps to strengthen the quality of the case study [23]. To increase reliability, data collection followed a case study protocol outlining the purpose and approach to the research. We relied on multiple sources of evidence (i.e., triangulation) and draft review by key informants to validate our data in relation to the aims of the study (i.e., construct validity).

The study was approved by the Regional Board for Ethical Vetting in Stockholm.

## Results

Results are organized in three sections: 1) the quantitative data and analysis; 2) the case description, including an overview of the care process pre-lean, the improvement process, and the content of the lean intervention; and 3) the case analysis based on the four lean principles.

### Performance outcomes

The ANOVAs reveal significant differences (p < 0.05) for both outcome variables and for the patient volume. The percentage of patients completing their visit and leaving the A&E within four hours increased from 67% pre-lean [95% CI (65,5: 69,7), N = 52] to 80% in the first year post-lean [95% CI (78,2:82,4), N = 52], a 19% increase. It remained at 80% [95% CI (78,3:82,6), N = 52] also in the second year post-lean. The average time to first physician consultation decreased from 67 minutes pre-lean [95% CI (61,7:71,5), N = 52] to 51 minutes in the first year post-lean [95% CI (46,5:56,3), N = 52], a 24% reduction. Results were sustained during the second year post-lean, at 54 minutes [95% CI (49,4:59,2), N = 52]. Meanwhile, the patient volume increased from 24,4 [95% CI (23,1:25,5), N = 52] visits per day (between 08.00 and 16.00) to 26,6 [95% CI (25,8:28,1), N = 52], a 9% increase and was 26,5 [95% CI (25:27,9), N = 52] in the second year post-lean.

**Figure 1:**
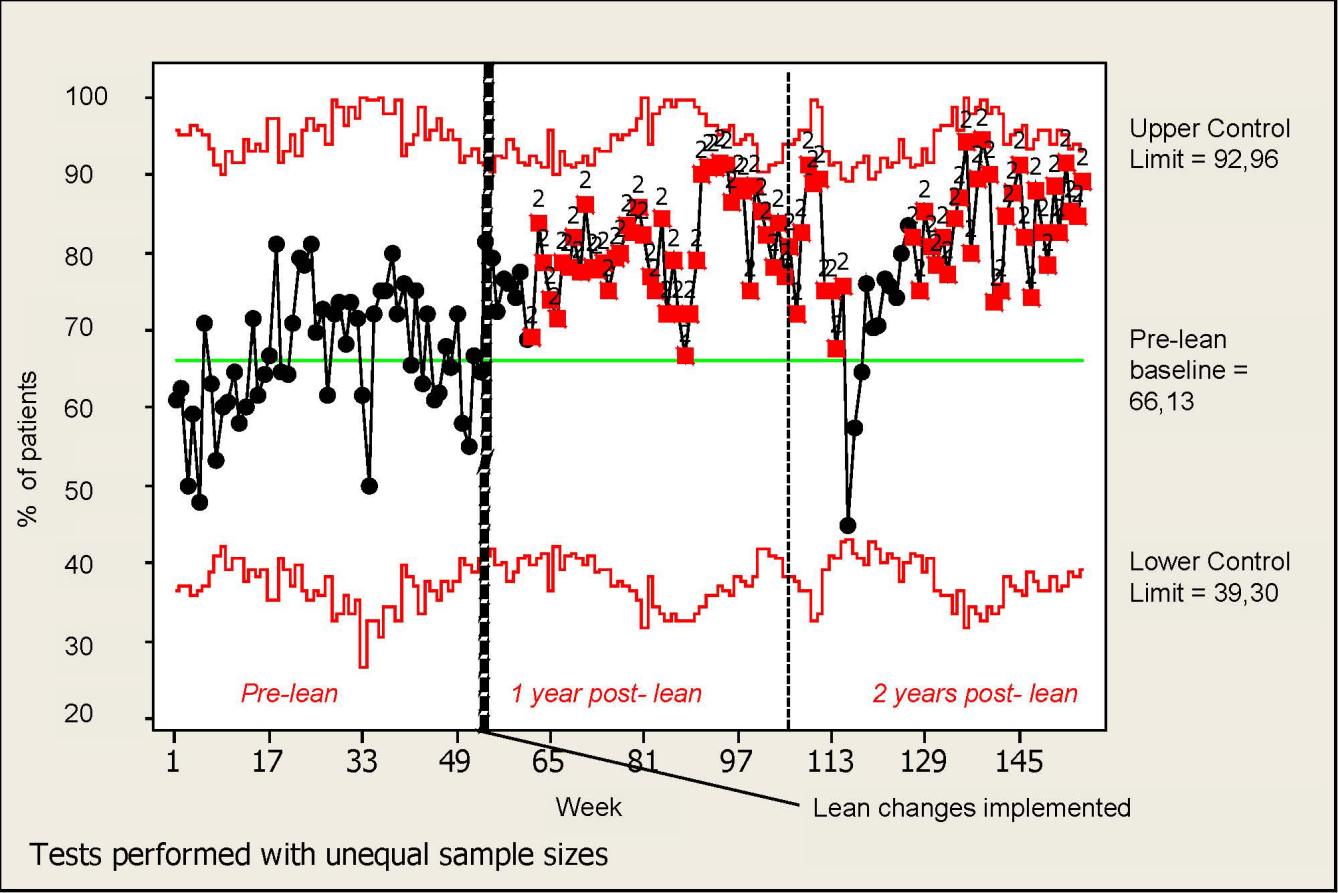
**P-control chart for percentage of patients leaving the A&E within four hours**. Special-cause variation is identified based on the decision rule: Nine consecutive points fall on the same side of the pre-lean baseline. The percentage of patients who leave the A&E within four hours pre-lean differs slightly from the value identified by the ANOVA as truncated values were used for the P-chart.

The P-chart in Figure 1 shows that a systematic increase in the percentage of patients who leave the A&E within four hours coincides with the implementation of the lean changes described below (see case description). The systematic change is indicated by nine consecutive points falling above the pre-lean baseline. According to the P-chart, the improvement is stable over time except for at approximately week 116.

**Figure 2:**
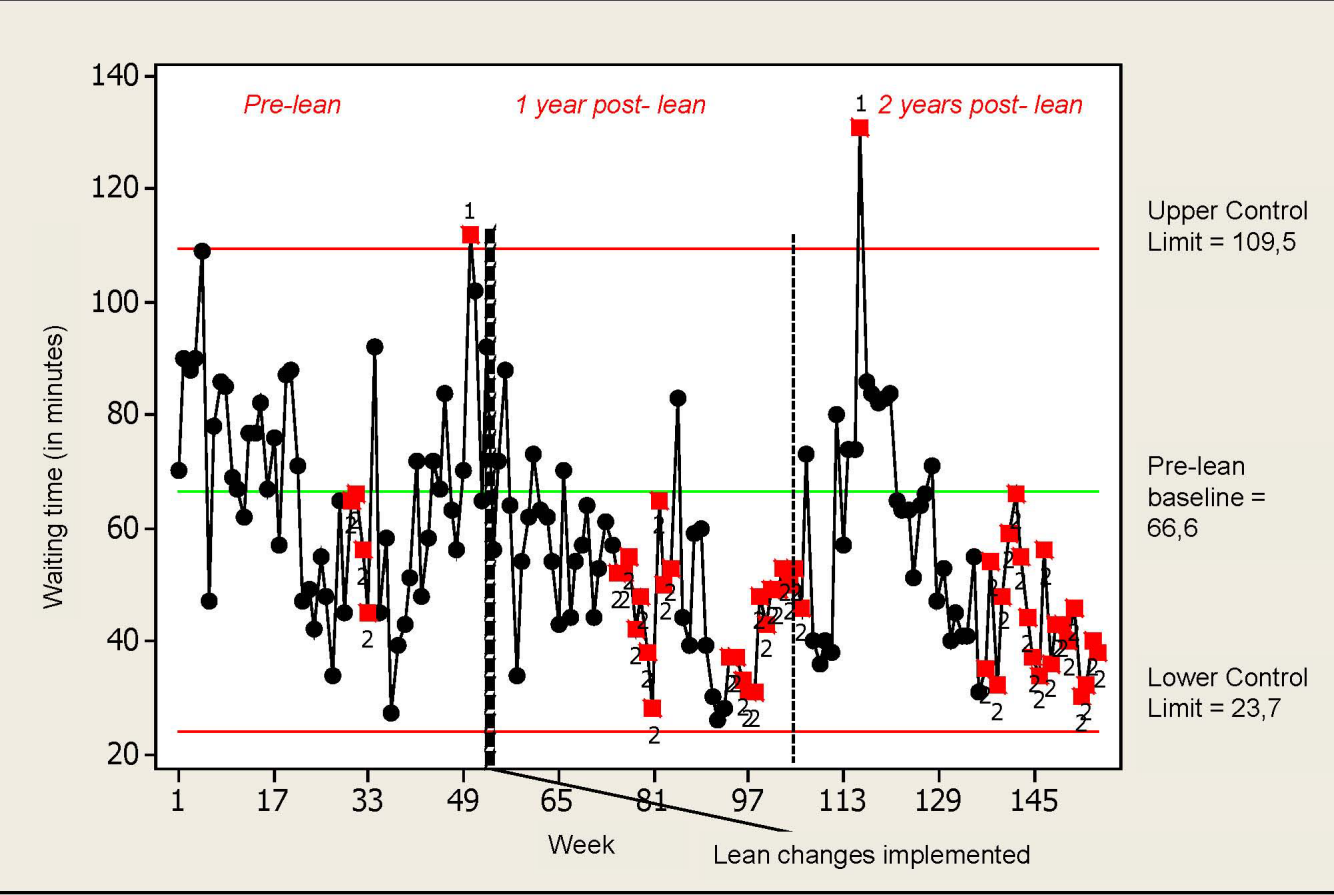
**I-chart for waiting time to see a physician**. Special-cause variation is identified based on the decision rules: Any single data point outside the 3σ limit; Nine consecutive points fall on the same side of the pre-lean baseline.

The I-control chart in Figure 2 indicates a systematic decrease of the waiting time to first physician assessment following week 53. The systematic change is indicated by several runs of nine consecutive data points falling on the same side of the pre-lean baseline. Significant improvements were also detected, based on the same rule, before the lean intervention, at weeks 30-33. There is also one case, during week 116, of a greater than 3σ-above baseline increase in waiting time followed by a steady return to lower waiting times.

### Case description

#### The pre-lean care process

Prior to the lean changes, patients were triaged upon arrival by a nurse (or by a physician if arriving by ambulance). Patients were prioritized based on a 1-5 scale triage score. This triage system had been locally developed to fit the characteristics and needs of pediatric patients, and was inspired by a triage system in use in Sweden named ADAPT (Adaptive Process Triage) [34]. The triage nurse entered patient information in an electronic health record. Patients were then sent to the waiting room or roomed directly, depending on clinical urgency and competing demand for care. A nurse printed the health record, placed it in the treatment area, and escorted the patient to a room. Assessment/treatment was initiated by physicians (usually residents) autonomously and at their own pace. When further investigation was needed physicians faxed referrals to other units and/or wrote orders on a paper chart which they then put in an "order box" at the nursing station. Any available nurse then acted on orders. When test results were ready, a nurse again placed the paper chart on the desk in the treatment area. On the physician's initiative the consultation continued until the patient was treated, admitted, or discharged. Often residents needed to consult a specialist, sometimes yielding further investigations or a change of plans. Since the A&E was usually staffed by only one senior physician-who, besides supervising residents, also answered phone calls from primary care, held seminars, took referrals, and made rounds on inpatient wards-such consultation typically delayed care further.

**Figure 3:**
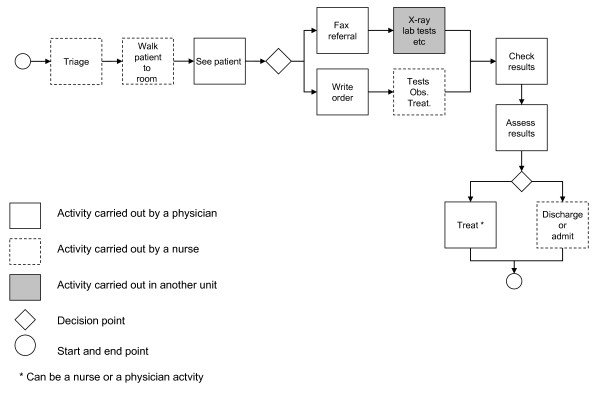
**Care process at the pediatric A&E**.

Figure 3 illustrates the main steps in the care process.

#### The improvement process

In the fall 2007, a recently qualified pediatrician (UB) was appointed to lead a team composed of nurses, nurse's aides, and physicians in redesigning the pediatric care flow at the Children's Hospital's A&E. The team was guided by a group of process improvement coaches, led by a senior cardiologist with extensive process improvement experience, who was appointed by the CEO. Together, they prepared the redesign by mapping the current process, identifying the patient inflow (expressed as the average number of patients, stratified by hour of arrival over a 24 h period), reviewing performance data and examining possible sources of waste-"non-value adding time". Waiting time to see a physician, both the initial physician contact and the follow-up assessment after the necessary investigations, were identified as the most important sources of waste.

Non-value adding waiting was found to occur due to a mismatch of capacity and demand. The team attributed this mismatch to: inefficient working procedures; shortage of senior physicians (the only senior physician on duty managed several competing tasks in parallel); employees engaged in multiple and simultaneous tasks which led to workflow interruptions; inadequate resources (number of staff members) and capacity planning (staff scheduling not matching typical demand patterns).

The coaches offered several Improvement Principles (Table 2), mainly drawn from lean thinking, to guide the team in their improvement work.

**Table 2:** Improvement principles used at the A&E

Principle	Description of the principle
Visualize	All people involved in the care process should have an overview of what happens, where one's colleagues are and where the patient is in the care chain.

Link patient care activities	The various activities that compose a patient's care process should, if possible, be linked together or even be performed in parallel.

Takt (work pace)	Different activities can take different time, but the goal is to decrease the variability in the time to complete each step in the process and to achieve a steady work pace to meet projected demand.

First-time quality	By getting things right the first time, quality is improved and the need for rework is reduced.

Standardize	To the extent possible, patient care processes should be standardized to reduce wasteful patient-to-patient variability.

Continual improvement	Processes and practices can be adjusted several times-by testing, evaluating, and trying again, using a scientific approach-before work flows smoothly

Hospital management established specific goals which were the same for all the hospital's acute care process flows: reduce the average time from patients' arrival until initial assessment by a physician to 40 minutes; and reduce the length of stay at the A&E so that 90% of all patients leave within 4 hours. The aim, in this initial phase, was to meet the goals between 08:00-16:00 on weekdays. Taking a stepwise approach to an "end-to-end" view on the patient "journey", process steps involving wards and support services such as radiology were left for later stages in the improvement effort.

Based on the Improvement Principles, and the best interest of patients, the improvement team proposed changes to help move from the "current state" to an "ideal state". The changes were summarized in a prototype of the new process. To design the prototype, the team started by looking at how work could be done to achieve a steady work pace, to make sure that the patient care activities were linked or carried out in parallel, and to get things right from the start. Secondly, the team suggested which competencies were needed and how staff could be protected from interfering tasks. Finally, they proposed which resources were necessary for this design to function.

The prototype was reviewed by the flow management group (the hospital CEO, the director of the Pediatric Division, and first-line managers) who concluded that the proposed changes were ready for testing. The team iteratively tested and modified them in three rounds. The tests, which involved the entire A&E, lasted 3-4 days and were conducted both between 08:00 and 16:00 and around the clock. The team evaluated the impact of the tests, informed by performance data and their own experiences.

After the third test, in November 2008, the management group approved the prototype, as modified through iterative tests, to be implemented as the standard way of working. The team, together with local managers, developed an implementation plan which was launched in December 2008.

#### The content of the change

The prototype that was eventually implemented had multiple components, described below.

##### Multi-professional care team approach and physical work setting redesign

Care delivery was reorganized around four parallel care teams per shift, each consisting of a physician (in most cases a resident) and a nurse or a nurse's aide. In contrast to the option adopted in other A&E areas at the hospital to always asses patients jointly, in this pediatric A&E patients were only seen by a physician and nurse together when necessary (usually urgent cases or patients with dyspnea), so as not to overwhelm pediatric patients with too many "white coats". In most cases, the physician examined the patient alone, and within a short time directly notified the nurse of further actions to be taken. To facilitate communication and information sharing, their work stations were moved next to one another so that they could work "at arm's length distance".

##### Centralized management and control of patient flow, and information technology

One specialist and one nurse on each shift were appointed as flow managers. Their roles were to assign patients to different doctor-nurse pairs based on the triage score previously assigned by the triage nurse, and on the availability and competence of each care team. No changes were brought to the priority system used for queuing. Flow managers planned patient entry to the treatment area in coordination with the four care teams. When they identified delays in a process they checked in with their colleagues and helped them out when needed. Care team members could also request assistance from the flow managers. To provide flow managers with an overview of the situation and facilitate their communication with team members, a flow manager work station was positioned in the middle of the treatment area. A computer monitor named "takt board" was set up to help flow managers appraise the flow pace (takt). The monitor showed, among other data, the hourly number of patients who had been initially assessed by a physician in relation to the expected pace. The expected unit work pace was calculated based on the average hourly patient inflow rate plus one standard deviation. Hence, some excess capacity, compared to average demand, was built into the system to absorb variability in patient in-flow. The monitor highlighted in red each hour during which the expected pace was not achieved by the unit. Then, a link appeared on the computer screen allowing flow managers to enter information about probable causes for the delay, in near real-time. This information was used retrospectively by the improvement team to make a root cause analysis as a basis for countermeasures.

##### Increased staffing and involvement of senior physicians

On the principle that the highest competence (i.e. competence needed to manage a case accurately and swiftly) should be involved at the earliest opportunity in the process, the specialist serving as flow manager discussed each patient case with more junior team physicians before the latter saw a patient. This consultation aimed to support each physician-nurse pair in their assessment and treatment efforts, and therefore to reduce uncertainties that might cause delays or unnecessary efforts. With less experienced team physicians, the specialist sometimes joined the team to see the patient, which rarely had happened in the past.

To protect the senior physicians from interfering tasks, one additional pediatrician was scheduled between 08:00 and 16:00 to serve as flow manager. The one pediatrician already allocated to the A&E was now assigned all the additional tasks, allowing the flow manager to focus on the care process and on supervising junior physicians.

##### Work schedule changes

Prior to the change, physicians usually stopped seeing new patients towards the end of a work shift to complete their administrative work (mostly clinical documentation) and thereby finish on time. This temporarily stopped the direct patient care flow and led to growing patient queues. To counteract this, physicians' work shifts were rescheduled to create overlap between shifts. After the change, incoming physicians saw new patients while outgoing physicians finished up their remaining patients and completed administrative tasks.

##### New roles and job descriptions

The new work arrangements entailed new organizational roles (e.g. nurse-physician care team members and flow managers). New roles and responsibilities were outlined and formalized in descriptions authorized by the department management.

##### Team approach to problem solving and continual improvement

After implementation, the improvement team, led by the process leader, continued their improvement efforts. At least every two weeks they reviewed performance together with the coach. Based on this and the Improvement Principles, they generated ideas which they tested and evaluated iteratively for subsequent implementation.

##### Monthly meetings with the management group

Every fifth week, the process improvement team, often represented by the process leader, met with the management group to report on progress (or lack thereof). Barriers to improvement were identified, as well as how and by whom these should be addressed. Managers decided which countermeasures to implement and follow up on and in this way participated in the improvement efforts.

### Case analysis: Understanding how and why the intervention worked and what may have prevented even greater improvement

While the activities that composed the care process remained the same after the intervention (Figure 3), changes were brought to the organization, management and improvement of the care process. Table 3 categorizes the intervention's components based on Spear and Bowen's principles [26]. The way in which each component worked in practice is analyzed below through the lenses of these four principles. The strengths and weaknesses of the intervention are also analyzed.

**Table 3:** Categorizing the intervention's [39] components using four lean principles

Lean principles	Intervention's components
Standardize work.	Specific new job roles-flow manager, team nurse and nurse's aide, and team physician-with job descriptions. Improvement Principles (IP)

Connect people that are dependent on one another.	Team-based organization and changes to work station location.

Create seamless, uninterrupted flow through the process.	Centralized management of the patient flow by flow managers, and changes in their work station location. Team-based organization and changes to work station location. Additional specialist staffing and work schedule changes.

Empower staff to investigate problems with the process and to develop, test, and implement countermeasures using a "scientific method".	Stable structures for continual improvement (team approach to problem solving and coach supervision, and takt board) involving also management (monthly meetings with the management group).

### Standardize work

Before the hospital-initiated improvement efforts, different actors assumed their roles and responsibilities based on spheres of expertise. While this approach is common in healthcare, it has been claimed to yield ambiguity about who should do what, when, and how [7,8]. The lean intervention brought new roles and responsibilities (flow managers, team nurse and nurse's aide, and team physician) which were further formalized in job descriptions. This contributed to reduce ambiguity and variation in how individuals carried out their work, as indicated by a nurse in an interview: *"[as a team nurse] one can more easily keep track of the patients one has ... I am responsible for my tasks, and for ensuring that they get done"*.

In addition, the hospital Improvement Principles facilitated a consistent, standard, and unambiguous way to work efficiently. Staff members referred to these Improvement Principles in their daily work and used them to continually improve the care process. As one nurse put it: "*we think in a different way now...we know how work is supposed to be done [at the A&E]"*.

On the other hand, these changes also led to some staff members feeling that their work was more narrowly regulated. Two nurses described the new approach as *"monotonous"*, and *"extremely driven by these key words [the Improvement Principles] that characterize the process"*.

### Connect people that are dependent on one another

Before lean, there was no explicit expectation concerning who should provide a service, to whom, and when. Thus, for example, any nurse available would act on a physician's order when they were free and felt ready to take on a new task. In addition, communication between care givers was asynchronous, mainly handled through paper charts left at the nursing station. In contrast, the team-based organization created clearer, synchronous connections between the caregivers involved in the process. A senior physician observed: *"Teamwork has improved ... there is a closer collaboration between professionals"*. Similarly a nurse stated "*when physicians need something, they know who to turn to, they know exactly which person they should talk to ... instead of asking [any of] the four nurses [at the A&E]"*. Coordination between care givers was also facilitated by the new work station arrangement, with care team members sitting together. As one nurse said: *"I know where my team physician is"*.

At the same time, inter-professional collaboration did not always work well. A resident said: "*It seems like nurses want to stay away... it is difficult to work together"*. Similarly, a nurse commented that: "*I would love to learn from doctors, but they are not all willing to cooperate and to have open communication!"*

### Create seamless uninterrupted flow through the process

Before the hospital-initiated improvement efforts, care givers shared responsibility for all patients at the A&E, in a rather implicit manner. Moreover, there was not explicit expectation for the timing of care providers' actions. With the changes, flow managers were explicitly assigned overall responsibility for work and patient flow at the A&E. A senior physician (working as a flow manager) noted that: "a*s a flow manager, one has a better overview of what is going on at the A&E"*. Similarly a flow nurse stated: "*before, everybody ran back and forth to act on physicians' orders, but nobody had an overview of what was going on at the A&E"*. Flow managers coordinated work with the goal of adhering to the new work patterns and were able to stop and address variances quickly. They also helped make individual tasks more standardized by assigning work to appropriate personnel and helping team members to coordinate. Flow managers were also able to assist residents in a timely manner and thereby enhanced the ability to get things right from the beginning without unnecessary delays and waste of resources. One pediatrician argued that: "*by discussing single cases with junior physicians at the beginning of the care process we have reduced the risk for unnecessary testing*". Or, as another pediatrician stated: "*flow managers have increased the productivity of junior physicians. Before they could take 2-3 patients during the whole day, because they could not move forward in the process ... they had no one to talk to*". Re-allocating a pediatrician position to serve as flow manager during the day shift, freed from other tasks, allowed the team physicians to work with fewer interruptions.

While flow managers seemed to contribute to more continuous work and patient- flow, some staff members raised concerns about being monitored. *"I believe there are people who dislike the new process as it makes it easier to track what each individual does" *said one senior pediatrician.

The team-based care approach also contributed to the achievement of simple and direct work and patient flows as it connected all care givers involved in a patient's care process. Indeed, some informants argued that the care team approach improved continuity of care as less people were involved in each case. Moreover, as team members were responsible to carry out all the work required to meet the needs of a designated patient, ambiguity was reduced. One nurse observed: "*There are fewer misunderstandings. There is no work duplication. One knows who has done what"*.

Some challenges concerning teamwork emerged. First, the ability to achieve flexible teams that would link all people involved in a care process was constrained by professional licensure regulations. For example, nurse's aides on the care team faced constraints on which tasks they were certified to perform. Thus, they were required to hand over some of their tasks to nurses on other teams, which resulted in perceived unevenness in workload between staff. Team-based work was also viewed by some nurses as *"too inflexible" *compared to the way work was organized before. A nurse stated: "*We [nurses and nurse's aides] have always worked as a team around all the patients and could be very flexible and it is really necessary because there are many situations in which we need to be more than one [nurse] as we are in the care team [now]*". In addition, new roles such as "team nurse" led to some frustration as nurses had to carry out tasks that did not require their professional qualification. One nurse said that: "*It feels a bit unnecessary [for me] to take three urine samples and a C-Reactive Protein test instead of doing things that you need to have a nurse for*".

The ability to achieve continuous flow through a team approach may also have been limited by the fact that patients were seldom assessed jointly by care team members, as the approach was not considered suitable for pediatric patients. Thus, the care process still consisted of a sequential approach with little, if any, parallel processing.

### Empower staff to investigate problems with the process and to develop, test, and implement countermeasures using a "scientific method"

The team approach to problem solving brought together members from different professions and helped them to understand how their work related to that of others and to patient needs. It also empowered people on the floor to manage processes and to come up with suggestions for improvement. Thus, the Improvement Principles introduced by improvement coaches could be translated by staff into process changes that fit their local context (e.g. the way the improvement group decided that team members should typically not see a new patient together from the start in order not to overwhelm patients with too many "white coats" all at once). The visual management system (takt board) was used to identify and document flow problems during the day, to support continual improvement. Guided by their coach, the process improvement team developed countermeasures that were consistent with the Improvement Principles. The coach summarized data and helped visualize results that were then fed back to the process improvement team to follow-up on results.

While successful in many ways, this approach to improvement also led to some frustration. Due to the large number of employees, including rotating staff, some clinicians (especially those not on the improvement team) felt they could not influence changes and were frustrated about the numerous modifications to the care process. Some also reported uncertainty about how work should be carried out. One nurse commented that: *"Changes occur too fast. If you miss a morning meeting, then you have missed the information!"*

Monthly meetings with the management group linked the problem solvers to the executive level with the information and authority needed to implement solutions that might otherwise have been beyond the reach of the improvement team, such as additional staffing or changes to work schedules. This helped open lines of communication across hierarchical levels in this large organization. As one physician stated: *"[It helps to] know that the issues and the wishes and the ideas we have for how we can develop this further will be discussed with the management group and that leaders show such a large commitment to this project"*.

## Discussion

This in-depth case study showed how major performance improvements-with reductions in patient waiting and lead times by 19-24%-were achieved and sustained over two years at a Swedish pediatric A&E following lean-inspired changes to employee roles, communication and coordination, expertise, schedule and staffing, workspace, and problem solving. These changes transformed operations by reducing individual work's ambiguity, creating clear connections between the caregivers who were dependent on one another, developing seamless and uninterrupted flow, and enabling continual improvement.

Before the hospital-initiated improvement efforts, operations at the A&E were characterized by unstable processes, unclear work methods, and a poor appreciation of demand and capacity. This situation is common in healthcare as operations are often not explicitly designed [17,35]. Like Radnor and Walley [17], we found the lean intervention to contribute to basic stability in operations. Lean promoted a process view, yielded more explicit work methods as well as roles and responsibilities, and enhanced stakeholders' understanding of capacity and demand. Lean also brought a structured approach to problem solving and linked improvement efforts to the hospital's strategy. Contributors to success included engaging healthcare professionals in designing, overseeing, and managing their own processes [35] and opening new lines of communication through the hospital hierarchy [36]. This is in line with previous research [37], and with Dickson et al.'s recent findings of the importance of frontline workers ownership and leadership commitment for successful lean implementation [38,39].

A lean-like program often contains many principles, tools, and practices [12,14,17]. Based on the work of Dean and Bowen [40], Åhsltröm defines "principles" as the "building blocks" of lean, and "practices" and "tools" as the activities undertaken to change operations [36]. Many articles and books have appeared all trying to reconstruct which principles really explain Toyota's high performance levels [10,41,42]. In contrast, contemporary research into lean healthcare mostly addresses which tools or practices worked, rather than developing a more general understanding of how or why lean works. Drawing on Spear and Bowen's principles (Table 1), this study adds to the current literature by explaining how lean worked in one specific paediatric A&E context based on Spear and Bowen's lean principles. While the specific changes described here may not necessarily be replicated by other A&Es, explanations offered here may be used to design change efforts to improve standardization, connect people, achieve continuous flow, and empower participatory problem solving with management involvement. To not include these building blocks in lean programs may result in inefficient or unsustainable approaches. For example, any process improvement program that includes process changes without incorporating an approach to continual improvement will likely fail in the long term [39]. As demonstrated in recent review articles, this is a risk many healthcare organizations face when they implement lean tools in isolation, or through a brief campaign [14,17].

While we found positive results in this pediatric A&E, Spear and Bowen's principles also helped us identify some contextual characteristics that might explain why even greater improvement was not achieved. The mismatch between job tasks, licensing constraints, and competence generated frustration among nurses and nurse's aides in relation to their work content and professional development. This mismatch also limited the ability to achieve flexible teams, something which, according to Åhlström, might challenge the implementation of lean in healthcare [36]. In the present case, this appeared also to limit the ability to achieve continuous flow. The perception of being monitored and discomfort with inter-professional collaboration also hindered further improvement. The large number of employees and the inevitable distance between some employees and change-related decision making led some employees to feel inadequately informed. These contextual issues indicate some possible challenges to lean's applicability in healthcare, compared with other sectors. Naturally, to fully realize the potential benefits of lean healthcare, organizations need to minimize the impact of such barriers and capitalize on facilitating conditions that are specific to their local context.

Some of the structural changes adopted by the case organization, including the care team approach and the development of new coordinating roles and responsibilities for nurses and physicians, have been described previously [43-45]. Unlike those described here, structural changes reported in prior literature were often combined with changes to the care process. Examples of related process changes reported in the literature include: immediate rooming of patients and bedside registration [43], test orders or other work conducted earlier in the process [43], and streaming patients in different patient flows [44,45]. The latter concerns the practice of grouping patients with similar processes, which has been demonstrated to reduce the risk for overcrowding [44-46]. These findings suggest that separating processes for patients likely to be discharged versus likely to be admitted might have been a useful complementary approach in the studied case.

### Methodological considerations and future research

While in-depth, this study has several important limitations.

The study lacked data to separate the impact of the lean intervention for patients who could go home directly versus those who were admitted. Such an analysis would have shown more precisely where the intervention was most effective. Indeed, inpatient admission bottlenecks can be expected to lead patients to spend more time than necessary at the A&E [47]. This also suggests the need for future research on how lean practices may address this important issue. In the studied A&E, efforts to improve the admission process had not yet begun at the time of data collection.

Due to the multi-component nature of the lean intervention, this study was unable to definitively attribute improvement to specific components of the intervention. While patient wait and lead times decreased-despite increasing volume-it is difficult to determine, for example, to what extent a specific change such as increased specialist staffing drove the improvements. Because no statistical test is available to disambiguate the effects of several changes applied concomitantly in a large-scale intervention, the case analysis sought to link specific changes to performance improvement using theorized lean principles. Taking an explanation building methodology [23], we used several sources of data to show that each intervention's component had a plausible effect on performance: reducing ambiguity, creating clear connections, developing seamless flow, or enabling continual improvement. Additionally, it is evident in the data that different components of the lean intervention interacted with one another. For example, increased specialist staffing occurred not as an isolated change but as a way to staff a new, lean-inspired flow manager position; similarly, this staffing increase may have been made possible by virtue of management's high level of support of the improvement group. Future research is needed to further disentangle which lean-inspired changes contribute the most to performance improvement. This will require creative study designs and strong theory in cases when interventions are implemented as a package rather than separated in time.

The use of previously developed theoretical principles is a means to generalization from single case studies [23]. Even so, further work should seek to support or modify the explanations offered here, through comparison to other single case studies or by applying a multiple-case study design [23]. Further work is also needed to support claims about the contextual conditions critical to further success and to assess the effects of lean interventions on employees' working conditions.

## Conclusion

Rather than merely arguing that "lean works" in healthcare, as most recent studied have done, we demonstrated how and why performance improvement resulted from a package of lean-inspired changes. We did this by applying four theoretical lean principles to demonstrate specific ways in which lean-inspired changes transformed work and improved performance in an A&E. The adapted lean principles offered here may enable healthcare organizations and managers to pick the right components of a lean program and to better understand the reasons behind lean's success (or failure).

## Competing interests

We hereby confirm that the article has been read and approved by all co-authors. UB has been part of the improvement team at the Astrid Lindgren Children's Hospital. All other authors declare that they have no competing interest and therefore have nothing to declare.

## Authors' contributions

PM, MB, HA, and JT, were involved in the original conception and design of the article.

MB obtained research funding. PM conducted data collection, and organized all data collected.

PM developed a case description based on data collected through interviews, observations, and document analysis. PM, RH, MB, HA, UB, and JT revised the case description. ME and PM conducted the statistical analysis. PM, RH, HA, UB, and JT contributed to the interpretation of the data presented under the heading "case analysis".

PM drafted the manuscript, and all authors contributed substantially to its revision. All authors have approved the final version of the paper. PM takes responsibility for the paper as a whole.

## Pre-publication history

The pre-publication history for this paper can be accessed here:

http://www.biomedcentral.com/1472-6963/12/28/prepub
